# Fault Diagnosis for Rotating Machinery Using Vibration Measurement Deep Statistical Feature Learning

**DOI:** 10.3390/s16060895

**Published:** 2016-06-17

**Authors:** Chuan Li, René-Vinicio Sánchez, Grover Zurita, Mariela Cerrada, Diego Cabrera

**Affiliations:** 1 School of Mechanical Engineering, Dongguan University of Technology, Dongguan 523808, China; 2 Department of Mechanical Engineering, Universidad Politécnica Salesiana, Cuenca 010105, Ecuador; rsanchezl@ups.edu.ec (R.-V.S.); grover_zurita@hotmail.com (G.Z.); cerradam@ula.ve (M.C.); dcabrera@ups.edu.ec (D.C.)

**Keywords:** fault diagnosis, deep learning, statistical feature, vibration sensor, rotating machinery

## Abstract

Fault diagnosis is important for the maintenance of rotating machinery. The detection of faults and fault patterns is a challenging part of machinery fault diagnosis. To tackle this problem, a model for deep statistical feature learning from vibration measurements of rotating machinery is presented in this paper. Vibration sensor signals collected from rotating mechanical systems are represented in the time, frequency, and time-frequency domains, each of which is then used to produce a statistical feature set. For learning statistical features, real-value Gaussian-Bernoulli restricted Boltzmann machines (GRBMs) are stacked to develop a Gaussian-Bernoulli deep Boltzmann machine (GDBM). The suggested approach is applied as a deep statistical feature learning tool for both gearbox and bearing systems. The fault classification performances in experiments using this approach are 95.17% for the gearbox, and 91.75% for the bearing system. The proposed approach is compared to such standard methods as a support vector machine, GRBM and a combination model. In experiments, the best fault classification rate was detected using the proposed model. The results show that deep learning with statistical feature extraction has an essential improvement potential for diagnosing rotating machinery faults.

## 1. Introduction

As one of the fundamental types of mechanical system, rotating machinery is widely applied in various fields. As a result of relative motion between mating surfaces, components of rotating machinery are prone to suffer from damage [1]. Effective fault diagnosis is thus important for maintaining the health of rotating machinery. One of the most challenging fault diagnosis tasks is the detection of faults and fault patterns, if any.

Different methods have been developed for fault diagnosis in rotating components such as gearboxes and bearings [2,3,4]. Gao *et al.* [5,6] systematically reviewed the fault diagnosis with model-based, signal-based, knowledge-based, and hybrid/active approaches. The most successful methods have three main steps: determining the fault symptoms, extracting the sensitive features, and classifying the condition patterns. Various fault symptoms, including vibration measurements [7], thermal features [8], acoustic signals [9], oil debris [10], and other process parameters have been used as indices of the health of rotating systems. Vibration sensor signals have been proven effective for monitoring the health of rotating machinery.

Even in the vibration sensor category, different features sensitive to fault detection have been extracted in recent years. Most of these feature extractions are performed in the time domain, frequency domain, and time-frequency domain. To extract a fault feature in the time domain, Raad *et al.* [11] proposed using cyclostationarity as an indicator to diagnose gears. A diagnostic feature was introduced by Bartelmus and Zimroz [12] to monitor planetary gearboxes in time-varying operating conditions. The fault features are sometimes very sensitive in the frequency domain. Spectral kurtosis is one of the most popular fault features in the frequency domain [13]. Based on frequency domain kurtosis, an optimal mathematical morphology demodulation method was proposed for the diagnosis of bearing defects [14]. Compared to feature extraction in the time and frequency domains, time-frequency domain features have attracted much attention in both academia and industry. Continuous wavelet transform (CWT) [15], discrete wavelet transform (DWT) [16], wavelet packet transform (WPT) [17], second generation wavelet transform [18], comblet transform [19], and other time-frequency tools [20,21] have been successfully used to generate fault-sensitive features. In addition to feature extraction in a single domain, researchers have proposed detecting machinery faults in different domains. Lei *et al.* [22] proposed two diagnostic parameters from an examination of the vibration characteristics of planetary gearboxes in both the time and the frequency domains.

Based on the extracted fault features, different classifiers have been used to distinguish the healthy condition from different fault patterns. A multi-stage feature selected by genetic algorithms was proposed by Cerrada *et al.* [23] for the fault diagnosis of gearboxes. An intelligent diagnosis model jointly using a wavelet support vector machine (SVM) and immune genetic algorithm (IGA) was introduced for gearbox fault diagnosis [24]. Discriminative subspace learning has been used to diagnose faults in bearings [25]. Tayarani-Bathaie *et al.* [26] introduced a dynamic neural network to diagnose gas turbine faults. An artificial neural network and empirical mode decomposition have been applied to automatic bearing fault diagnosis using vibration signals [27]. It is clear that the SVM family has achieved good results in comparison with peer classifiers. Recently, deep learning has gained much attention in the classification community. Tamilselvan and Wang [28] introduced deep belief learning based health-state classification for failure diagnosis in datasets including iris, wine, Wisconsin breast cancer diagnosis, *Escherichia coli* and others. Tran *et al.* [29] used deep belief networks for the diagnosis of reciprocating compressor valves.

In this paper, we present a deep statistical feature learning approach for fault diagnosis in rotating machinery. The purpose of this paper is to use deep statistical feature learning as an integrated feature optimization and classification tool to improve fault diagnosis capability. For deep learning of statistical features with unknown value boundaries, a Gaussian-Bernoulli deep Boltzmann machine (GDBM) based on Gaussian-Bernoulli restricted Boltzmann machines (GRBMs) is proposed for the automatic learning of fault-sensitive features. The influences of different domains and typical rotating mechanical systems on fault classification are investigated. Deep learning is an effective learning framework for simultaneous statistical feature representation and classification, and the GRBM is a promising tool for dealing with unknown-boundary problems within the deep learning framework.

The remainder of this paper is structured as follows: the statistical features of the machinery vibration measurements are introduced in Section 2, and feature learning using the unsupervised GRBM and the supervised GDBM are also proposed in this section. In Section 3, fault diagnosis experiments for a gearbox and bearings are reported. The results of the experiments and discussions of the results are presented in Section 4. Conclusions are given in Section 5.

## 2. Methodologies

The GDBM is applied as a deep statistical feature learning tool for fault diagnosis in this paper. The methodologies used are introduced in this section. In Section 2.1, some classical statistical features are calculated from the time, frequency, and time-frequency domains of the vibration measurements. As the GDBM is constructed by stacking several GRBMs, and the GRBM is an improved version of the restricted Boltzmann machine (RBM), in Section 2.2 the basics of the GRBM are introduced. The statistical features calculated in the first subsection are used as the fault features represented by the unsupervised GRBM. As deep learning is an effective learning framework for simultaneous statistical feature representation and classification, the GDBM is constructed in Section 2.3. More details can be found in the following sections.

### 2.1. Statistical Features of the Vibration Sensor Signals

For a vibration measurement *x*(*t*) of the rotating machinery, its spectral representation *X*(*f*) can be calculated by:

(1) $$X(f)=\hat{x}(f)=\int_{-\infty }^{+\infty }x(t)e^{-2\pi jft}dt$$

where the hat “^” stands for the Fourier transform, *t* the time and *f* the frequency. For engineering applications, the collected vibration data are discrete values. Hence, the discrete version of Equation (1) (*i.e.*, the discrete Fourier transform, DFT) should be used for the vibration data. There are several ways to calculate the DFT, among which the fast Fourier transform (FFT) is an efficient solution.

The time domain measurement *x*(*t*) and the frequency domain spectrum *X*(*f*) are capable of describing the machinery vibration in terms of time and frequency separately. For jointly representing the machinery vibration, the wavelet transform provides a powerful mathematical tool for signal processing and analysis. As mentioned in the Introduction, the CWT, DWT and WPT are in general the most popular categories in the wavelet transform family. Although different wavelet transforms have been successively applied in the fault diagnosis community, this paper uses the WPT to generate the time-frequency statistical features because it has comparatively low dimensions of the decomposition numbers and enhanced signal decomposition capability in the high frequency region.

The WPT is an extension of the typical DWT, in which detailed information is further decomposed by the WPT in the high frequency region. In other words, the WPT decomposes *x*(*t*) into a set of wavelet packet (WP) nodes through a series of low-pass and high-pass filters recursively. 

With the integral scale parameter *j* and translation parameter *k* (*k* = 0, …, 2*^j^* − 1; *j* = 0, …, *J*, which is the number of the decomposition levels), a WP function $T_{j,k}^{n}(t)$ is defined by:

(2) $$T_{j,k}^{n}(t)=2^{j/2}T^{n}(2^{j}t-k)$$

where *n* = 0, 1, … is the oscillation parameter [30]. The first two WP functions with *j* = *k* = 0 are the scaling function *ϕ*(*t*) and the mother wavelet function *ψ*(*t*), respectively. The remaining WP functions for *n* = 2, 3, … can be given by the WPT as:

(3) $$T^{2n}(t)=\sqrt{2}\sum_{k}h(k)T_{1,k}^{n}(2t-k)\text{ and }T^{2n+1}(t)=\sqrt{2}\sum_{k}g(k)T_{1,k}^{n}(2t-k)$$

where the low-pass filter *h*(*k*) and the high-pass filter *g*(*k*) have the following forms:

(4) $$h(k)=\frac{1}{\sqrt{2}}<\varphi (t),\varphi (2t-k)>\text{ and }g(k)=\frac{1}{\sqrt{2}}<\psi (t),\psi (2t-k)>$$

where <*,*> represents the inner product operator. The WP coefficients $P_{j,k}^{n}$ are therefore the inner product between the signal and the WP functions, *i.e.*:

(5) $$P_{j,k}^{n}=<x(t),T_{j,k}^{n}>=\int_{-\infty }^{\infty }x(t)T_{j,k}^{n}(t)d(t)$$

In this way, the signal *x*(*t*) is decomposed by the WPT into *J* levels. At the *j-*th (*j* = 0, …, *J*) level, there are 2*^j^* packets with the order *n* = 1, 2, …, 2*^j^*. For simplicity, we index the WP node as (*j*, *n*) whose coefficients are given by $P_{j,k}^{n}$.

According to the above analyses, the vibration measurement of the rotating machinery can be represented in the time domain, the frequency domain and the time-frequency domain. This can be formulated by:

(6) $$M(p,q)=\left\{ \begin{array}{ll} x(t); & p\in R^{1},q=t\in R^{n_{0}},\text{time domain}\\ X(f); & p\in R^{1},q=f\in R^{\left\lfloor n_{0}/2\right\rfloor },\text{frequency domain}\\ {}[P_{1,k}^{1},P_{1,k}^{2},...,P_{j,k}^{2^{j}}]; & p\in R^{2^{J+1}-1},q\in R^{n_{0}/2^{j}},\text{time-frequency domain} \end{array}\right.$$

where *n*_0_ is the length of *x*(*t*).

As the three representations *M*(*p*,*q*) are usually very long, statistical features can be used as healthy condition indicators for rotating machinery. Statistical features have been approved as simple and effective features in fault diagnostics [17]. Based on the aforementioned studies, one can use the following statistical features for the vibration signals:

(7) $$\begin{array}{l} F_{1,p}(M)=\frac{\int_{-\infty }^{\infty }{\left[M-\mu \right]}^{4}P(M)dM}{\sigma^{4}},F_{2,p}(M)=\frac{\int_{-\infty }^{\infty }{\left[M-\mu \right]}^{3}P(M)dM}{\sigma^{3}},\\ F_{3,p}(M)=\frac{\max|M|}{\sqrt{\frac{1}{N}\sum_{q=1}^{N}M^{2}}},F_{4,p}(M)=\frac{\max|M|}{{\left(\frac{1}{N}\sum_{q=1}^{N}\sqrt{\left|M\right|}\right)}^{2}},F_{5,p}(M)=\frac{\sqrt{\frac{1}{N}\sum_{q=1}^{N}M^{2}}}{\frac{1}{N}\sum_{q=1}^{N}\left|M\right|},\\ F_{6,p}(M)=\frac{\max|M|}{\frac{1}{N}\sum_{q=1}^{N}\left|M\right|},F_{7,p}(M)=\int_{-\infty }^{\infty }{\left[M-\mu \right]}^{2}P(M),F_{8,p}(M)={\left(\frac{1}{N}\sum_{q=1}^{N}\sqrt{\left|M\right|}\right)}^{2}\text{, and}\\ F_{9,p}(M)=\frac{1}{N}\sum_{q=1}^{N}\left|M\right| \end{array}$$

where *N* is the length of *q* for *M*(*p*,*q*), *P*(.) is the probability density [31], *µ* is the mean value, *σ* is standard deviation, and *F*_1,*p*_, …, *F*_9,*p*_ stand for kurtosis, skewness factor, crest factor, clearance factor, shape factor, impulse indicator, variance, denominator of clearance factor (the square of the averaged square roots of absolute amplitude), and mean of absolute amplitude values of the *p-*th vector of *M*(*p*,*q*), respectively [32]. Note that there are nine statistical features for the time domain representation *M*(*p*,*q*) = *x*(*t*), 9 for the frequency domain representation *M*(*p*,*q*) = *X*(*f*), and 9(2*^j^* ^+^^1^ − 1) for the time-frequency domain representation *M*(*p*,*q*) = $\left[P_{1,k}^{1},P_{1,k}^{2},...,P_{j,k}^{2^{j}}\right]$. The feature set *F* is therefore given by:

(8) $$F=\left\{ \begin{array}{ll} {}[F_{1,1}(M),...,F_{9,1}(M)]; & \text{time domain}\\ {}[F_{1,1}(M),...,F_{9,1}(M)]; & \text{frequency domain}\\ {}[F_{1,1}(M),...,F_{9,1}(M),F_{1,2}(M),...,F_{9,2}(M),...,F_{9,2^{J+1}-1}(M)]; & \text{time-frequency domain} \end{array}\right.$$

### 2.2. Statistical Feature Representation by Unsupervised Boltzmann Machines

After determining the statistical features in the time domain, the frequency domain and the time-frequency domain, in this subsection the unsupervised Boltzmann machine is proposed for feature representation.

The deep learning is a promising branch of the machine learning. It was developed to simulate the working mechanism of the brain to make sense of such data as images, sounds, and texts. The composed single layer GRBM model is the core to construct the deep learning (GDBM) frameworks in this work, and is originated from restricted Boltzmann machine (RBM).

The Boltzmann machine is a log-linear energy based model, where the energy function is linear in its free parameters. To restrict the Boltzmann machines to those without visible-visible and hidden-hidden connections, the RBM was proposed by Hinton, the father of deep learning, to form deep learning networks [33]. 

Conventional RBMs define the state of each visible and hidden neuron as binary codes (0 or 1). For real-valued data, the RBM has to normalize the input variables into [0, 1] with treating them as probabilities. For regular cases where the real values data have limited values, e.g., [0, 255] for pixels in the image processing, the RBM works well [34]. However, our statistical features scatter in different ranges. For example, the minimal value for *F*_1_ is 0, but that for *F*_5_ will be a negative number. This means that the conventional RBM is difficult to cope with our statistical features for the fault diagnosis.

**Figure 1:**
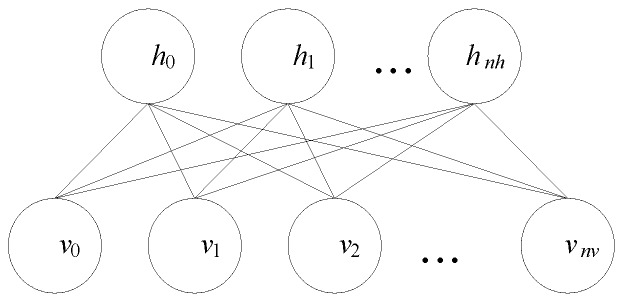
Illustration of the network connections with a GRBM. Note the GRBM exhibits same structure compared to its RBM counterpart.

To accommodate the real-valued data, the binary visible neurons can be replaced by the Gaussian ones to generate the Gaussian-Bernoulli RBM (GRBM). Although with real-valued neurons, the GRBM exhibits same structure compared to its RBM counterpart as shown in Figure 1.

For the GRBM shown in Figure 1, the energy function *E*(**v**, **h**) is given by:

(9) $$E(v,h|\theta )=\sum_{i=1}^{n_{v}}\frac{{\left(v_{i}-b_{i}\right)}^{2}}{2\sigma_{i}^{2}}-\sum_{i=1}^{n_{v}}\sum_{j=1}^{n_{h}}W_{ij}h_{j}\frac{v_{i}}{\sigma_{i}^{2}}-\sum_{j=1}^{n_{h}}c_{j}h_{j}$$

where **v** and **h** denote the visible and the hidden neurons, *b_i_* and ***c****_i_* stand for the offsets of the visible layers, *w_ij_* represents the weights for the connection matrix, *σ_i_* is the standard deviation associated with a Gaussian visible neuron *v_i_*, and *θ* is the Gaussian parameter [35]. The traditional gradient-based training of the GRBM has difficulty learning *σ_i_*, which is constrained to be positive. Hence, some algorithms fix *σ_i_* as unity. With the improved energy function, Cho *et al.* [35] proposed conditional probabilities for the visible and the hidden neurons as follows:

(10) $$p(v_{i}=v|h)=Ν(v|b_{i}+\sum_{j=1}^{n_{h}}h_{j}w_{ij},\sigma_{i}^{2})$$

and

(11) $$p(v_{i}=v|h)=Ν(v|b_{i}+\sum_{j=1}^{n_{h}}h_{j}w_{ij},\sigma_{i}^{2})\;\mathrm{and}\;p(h_{i}=1|v)=S(c_{j}+\sum_{i=1}^{n_{v}}w_{ij}v_{i}/\sigma_{i}^{2})$$

where $Ν(.|\mu ,\sigma^{2})$ is the Gaussian probability density function with mean *μ* and variance *σ*^2^, and *S*(.) is a sigmoid function. The upgraded gradients with respect to the GRBM parameters are given by:

(12) $$\nabla T_{ij}={\langle v_{i}h_{j}/\sigma_{i}^{2}\rangle }_{d}-{\langle v_{i}h_{j}/\sigma_{i}^{2}\rangle }_{m},\;\nabla b_{i}={\langle v_{i}/\sigma_{i}^{2}\rangle }_{d}-{\langle v_{i}/\sigma_{i}^{2}\rangle }_{m},\;\nabla c_{j}={\langle h_{j}\rangle }_{d}-{\langle h_{j}\rangle }_{m},\;\nabla \log\sigma_{i}^{2}=\exp(-\log\sigma_{i}^{2})\left({\langle {\left(v_{i}-b_{i}\right)}^{2}/2-\sum_{j=1}^{n_{h}}v_{i}h_{j}w_{ij}\rangle }_{d}-{\langle {\left(v_{i}-b_{i}\right)}^{2}/2-\sum_{j=1}^{n_{h}}v_{i}h_{j}w_{ij}\rangle }_{m}\right)$$

where <.>_d_ and <.>_m_ represent the expectation computed over the data and the model distributions, respectively.

When applying the GRBM for the unsupervised learning of the statistical features, the feature set *F* should be used as **v** and the GRBM results *GR*(*F*) = **h**. In this way, the *n_v_* statistical features are represented by *n_h_* neurons [36]. For condition monitoring and fault type classification, GRBM representations can be input to a classifier such as a support vector machine (SVM), decision tree, or random forest.

When applying the SVM as a classifier for fault diagnosis in rotating machinery, one should choose a multi-class SVM. The classical SVM is a binary classifier. Different methods have been proposed for using classical SVMs to compose multi-class SVMs. A pairwise coupling strategy was introduced by Hastie and Tibshirani [37] to perform multi-class classification by combining posterior probabilities provided by individual binary SVM classifiers.

### 2.3. Deep Statistical Feature Learning and Classification

After determining the statistical features in the time domain, the frequency domain and the time-frequency domain, in this subsection the unsupervised Boltzmann machine is proposed for feature representation.

In a common sense, an unsupervised mono-layer GRBM is inferior to a supervised multi-layer deep model. To stack several GRBMs on top of each other, a Gaussian-Bernoulli deep Boltzmann machine (GDBM) can be constructed for deep statistical feature learning of the machinery vibration signals. As an extension of the classical deep Boltzmann machine (DBM), the GDBM was introduced by Cho *et al.* [36]. Unlike other RBM-based deep models such as the deep belief network and the deep autoencoder, each neuron in the intermediate layers of the GDBM connects with both top-down and bottom-up information.

**Figure 2:**
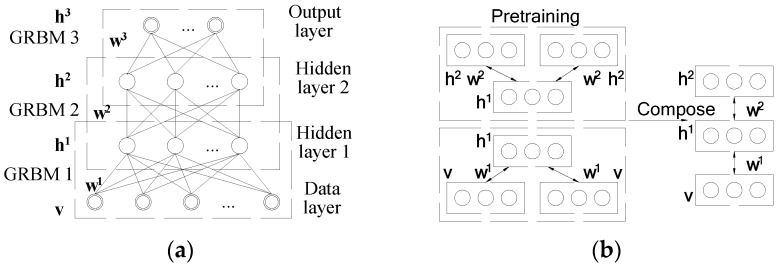
Schematic of the three-layer GDBM: (**a**) network structure; and (**b**) pretraining and composition of the GDBM.

The GDBM structure used in this paper is shown in Figure 2a. The suggested GRBM is composed of three GRBMs (*i.e.*, GRBM_1_, GRBM_2_, and GRBM_3_). Each GRBM consists of one visible layer and one hidden layer, and the hidden layer of the previous GRBM is just the visible layer of the next GRBM. In this way, the first layer (data layer) and the second layer (hidden layer 1) forms the GRBM_1_, the second layer and the third layer (hidden layer 2) forms the GRBM_2_, the third layer and the last layer (output layer) forms the GRBM_3_, and the three GRBMs are stacked together to form the GDBM.

The GDBM and its constituting GRBMs can be pretrained using a greedy, layer-by-layer unsupervised learning algorithm [37]. During the pretraining period as shown in Figure 2b, special attention should be paid to the GDBM as the neurons in the intermediate layers receive information both from the upper and the lower layers. To cope with this particularity, Salakhutdinov [38] halved the pretrained weights in the intermediate layers and duplicated the visible and topmost layers for the pretraining. With this idea, Equation (10) should be revisited to calculate the energy of the visible layer for the GRBM as:

(13) $$E(v,h^{\left(1\right)}|\theta )=\sum_{i=1}^{n_{v}}\frac{{\left(v_{i}-b_{i}\right)}^{2}}{2\sigma_{i}^{2}/N_{v}}-\sum_{i=1}^{n_{v}}\sum_{j=1}^{n_{h}}w_{ij}h_{j}^{\left(1\right)}\frac{v_{i}}{\sigma_{i}^{2}N_{v}}-\sum_{j=1}^{n_{h}}c_{j}h_{j}^{\left(1\right)}$$

where *N_v_* = 2 corresponds to the duplication of the visible layer. Similarly, the energy for the topmost GRBM*_L_* during the pretraining is given by:

(14) $$E(h^{\left(L-1\right)},h^{\left(L\right)}|\theta )=-\sum_{j=1}^{n_{h}}N_{v}c_{j}h_{j}^{\left(L\right)}-\sum_{i=1}^{n_{v}}\sum_{j=1}^{n_{h}}N_{v}w_{ij}^{\left(L-1\right)}h_{i}^{\left(L-1\right)}h_{j}^{\left(L\right)}-\sum_{i=1}^{n_{v}}b_{i}h_{i}^{\left(L-1\right)}$$

The aforementioned pretraining is an unsupervised, bottom-up procedure for the GDBM. This means that it cannot be applied for the classification after the pretraining. Compared to conventional unsupervised learning, fortunately, the GDBM requires an extra supervised, top-down fine-tuning procedure [39,40]. At the fine-tuning procedure, the output layer is replaced by a multilayer perceptron (MLP) with sigmoid functions. To fit the fault classification task, all the weights **w** can be discriminatively fine-tuned using a back-propagation (BP) algorithm [41]. The supervised BP method uses labeled data as an extra MLP layer of variables to train the GDBM model for the classification. Unlike the unsupervised training process considering one GRBM at a time, the BP training considers all the layers in a GDBM simultaneously, which is in the same way as for the standard feed forward neural networks [42]. In this way, the GDBM can be regarded as an improvement of the MLP, or neural networks. It is capable of dealing with the classification for nonlinear, abnormal (non-Gaussian) data using a “deeper” fashion [43]. Of course, this “deeper” learning is much more time-consuming than the conventional ones.

**Figure 3:**
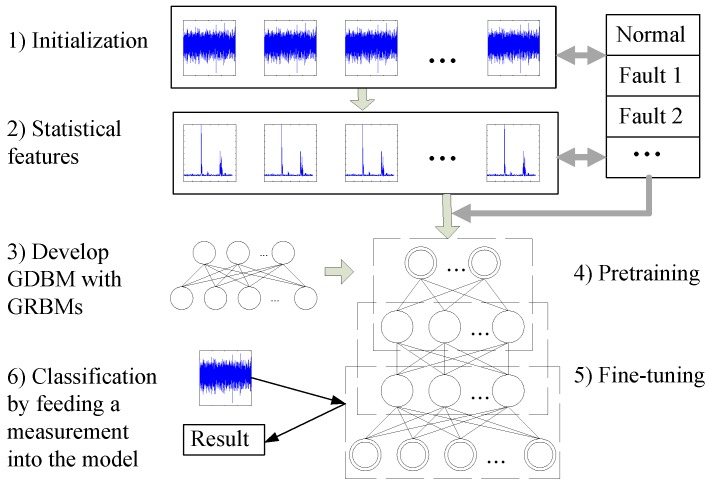
Flowchart of the deep statistical feature learning technique for the fault diagnosis of the rotating machinery.

Having introduced the GDBM and its constituting components, the GRBMs, the procedure of applying the GDBM based classification for the fault diagnosis of the rotating machines is shown in Figure 3 and is summarized as follows:

Step 1. Collect the vibration signals *x*(*t*), define the fault patterns and the diagnosis problems;
Step 2. Calculate the statistical feature set *F* according to Equation (8);
Step 3. Develop the GDBM model with the stack of the GRBMs according to Figure 1 and Figure 2;
Step 4. Pretrain the GDBM model and its constituting GRBMs using the layer-by-layer unsupervised learning algorithm from the training dataset;
Step 5. Fine-tune the GDBM weights using the BP algorithm from the training dataset; and
Step 6. Diagnose the rotating machinery condition using the trained GDBM model.

## 3. Data Collection Experiments for the Fault Diagnosis

To validate the effectiveness of deep statistical feature learning for fault diagnosis, the proposed deep learning was applied to diagnose the health of two rotating mechanical systems. The experimental setups and procedures are detailed in the following two subsections.

### 3.1. Experimental Procedure for Gearbox Fault Diagnosis

**Figure 4:**
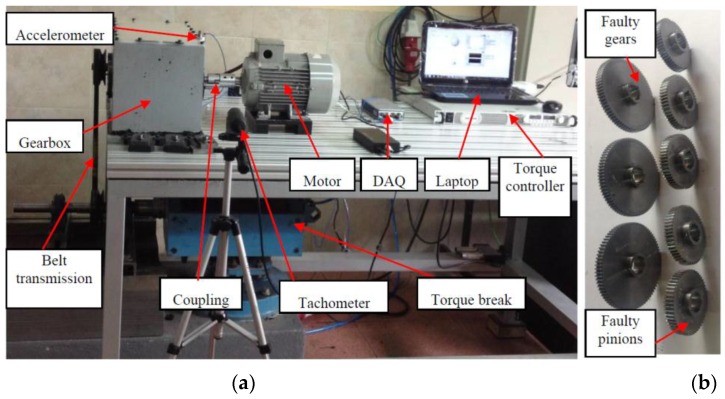
Gearbox fault diagnosis configurations: (**a**) experimental set-up; and (**b**) three different faulty gears and five different faulty pinions.

The first experiments were carried out on a gearbox fault diagnosis system. As shown in Figure 4a, the output of a motor (3~, 2.0 HP, Siemens, Munich, Germany) was connected to the input shaft of a gearbox (fabricated by the lab of the Universidad Politécnica Salesiana, Cuenca, Ecuador) via a coupling. A 53-tooth pinion was installed on the input shaft of the gearbox, whose output shaft has an 80-tooth gear. An electromagnetic torque break (8.83 kW, Rosati, Monsano, Italy) was used as a load to connect with the output shaft of the gearbox via a belt transmission. The torque break was controlled by a controller (GEN 100-15-IS510, TDK-Lambda, Tokyo, Japan) which enabled the load to be adjusted manually. An accelerometer (ICP 353C03, PCB, Depew, NY, USA) was mounted on top of the gearbox to collect the vibration signals, which were sent to a laptop (Pavilion g4-2055la, HP, Palo Alto, CA, USA) through a data acquisition system (cDAQ-9234, NI, Austin, TX, USA). The laptop controlled an inverter (VLT 1.5 kW, Danfoss, Nordberg, Denmark) for adjusting the motor’s rotation speed, which was monitored by a tachometer (VLS5/T/LSR optical sensor, Compact, Bolton, UK).

In the gearbox fault diagnosis experiments, in addition to one normal pinion and one normal gear, three different faulty gears and five different faulty pinions (shown in Figure 4b) were used to configure different condition patterns for the gearbox. The 10 different patterns shown in Table 1 were set for the collection of vibration signals. 

To challenge the fault diagnosis performance, three different load conditions (no load, small load, and large load), were manually set for each pattern. For each pattern and load condition, we collected 24 signals, each of which covered 0.4096 s, with a sampling frequency of 10 kHz. The experiments were repeated five times, so 3600 vibration signals corresponding to 10 condition patterns (with three different loads) were recorded. Each vibration signal was used to generate the temporal, spectral, and WPT representations *M*(*p*,*q*) given by Equations (6)–(8) were then used to generate the feature set *F* for the vibration signals. The 3600 feature sets were divided into a training dataset with 2400 samples and the testing dataset with 1200 samples.

The unsupervised GRBM and the supervised GDBM were applied to learn the statistical features of the vibration signals. The statistical features represented by the unsupervised GRBM required an additional classifier for the pattern classification. Considering its excellent classification capability, the SVM was used as the classifier for the GRBM representations. For the GDBM, supervised deep learning as shown in Figure 3 was applied for the healthy condition pattern classification of the gearbox.

### 3.2. Experimental Procedure for Bearing Fault Diagnosis

To further challenge the deep statistical feature learning for fault diagnosis, we also carried out bearing fault diagnosis experiments. The gear fault patterns (displayed in Table 1) occupied areas of great damage, which introduced greater changes in the vibration measurements [44]. Compared to the vibration signal of the gear fault, an incipient bearing fault often has a smaller damage surface and thus generates weak vibration changes [45].

**Figure 5:**
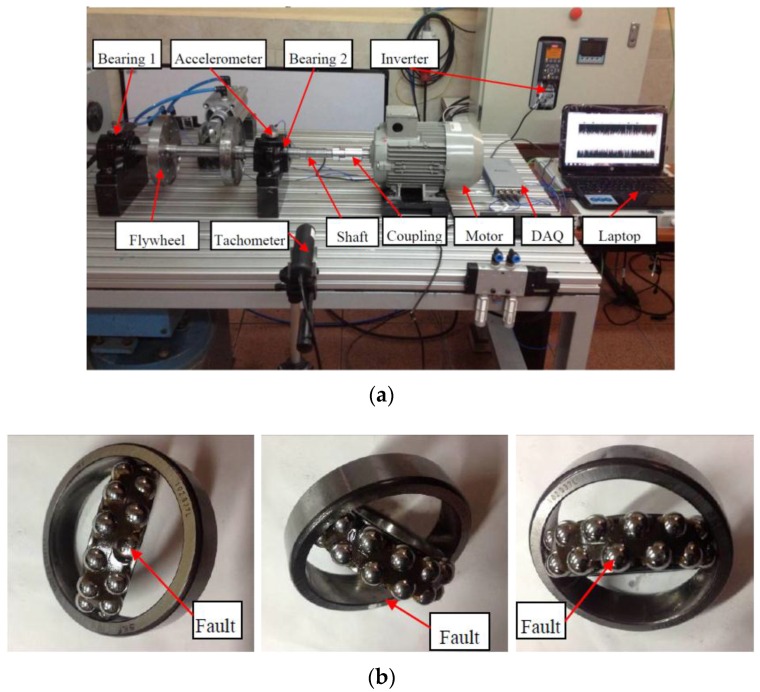
Fault diagnosis configurations for the rolling element bearings: (**a**) experimental set-up; and (**b**) 3 different faulty bearings with an inner race fault (**left**), an outer race fault (**middle**) and a ball fault (**right**), respectively.

As shown in Figure 5a, a rolling element bearing test rig was constructed in the Universidad Politécnica Salesiana of Ecuador to collect the vibration measurements for different healthy conditions. The test rig was driven by a motor (3~, 2.0 HP, Siemens) controlled by an inverter (VLT 1.5 kW, Danfoss). The rotating speed of the motor was monitored by a tachometer (VLS5/T/LSR optical sensor, Compact). A steel shaft (ϕ30 mm) was connected to the motor via a coupling. The two ends of the shaft were supported by two bearings (bearing 1 and bearing 2, 1207 EKTN9/C3, SKF, Goteborg, Sweden). An accelerometer (ICP 353C03, PCB) was mounted on the housing (SNL 507-606, SKF) of bearing 2 for measuring the vibration signals, which were collected by a data acquisition box (cDAQ-9234, NI) that communicated with a laptop (Pavilion g4-2055la, HP). Two flywheels were installed on the shaft as the load of the system.

In addition to the normal bearings, as shown in Figure 5b, three different faulty bearings with an inner race fault, an outer race fault and a ball fault, were used in the experiments. Using combinations of bearings in different conditions, seven healthy condition patterns were set, as shown in Table 1. For each experiment with each pattern, there were respectively 0, 1 and 2 flywheels used as the load. For each pattern and load configuration, 48 signals were collected for 0.4096 s. Each experiment was repeated five times. This means that 5040 signals were finally obtained. The sampling frequency for the bearing fault diagnosis was also set at 10 kHz.

Similar to the procedure described in the previous section, the statistical features were produced from the raw data of the bearing vibration signals. The unsupervised GRBM and the supervised GDBM were again applied for the fault diagnosis of the bearing system. The results of all the experiments are detailed in the next section.

## 4. Results and Discussion

### 4.1. Gearbox Fault Diagnosis Results

**Figure 6:**
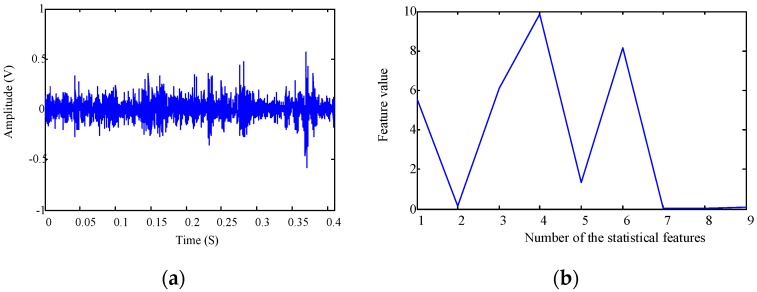

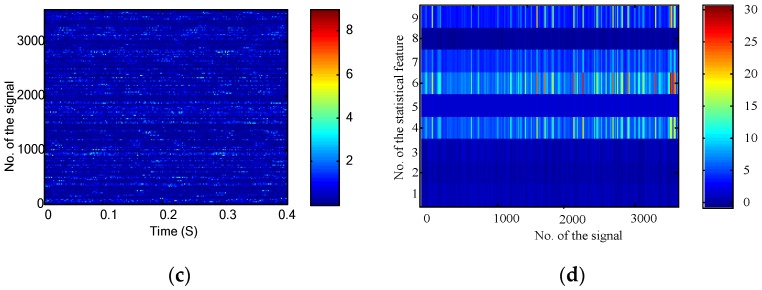
Time domain features for the gearbox fault diagnosis: (**a**) time domain waveform of the first signal; (**b**) time domain statistical features of the first signal; (**c**) time domain waveforms of the 3600 collected signals; and (**d**) time domain statistical features of the 3600 collected signals.

Figure 6a,b plot the time domain waveform and statistical features for the first signal collected from the gearbox experimental setup. As the signal covered 0.4096 s with the sampling frequency of 10 kHz, the length of the discrete time signal is 4096. For all the collected 3600 signals, their time domain waveforms and statistical features are shown in Figure 6c,d, respectively.

**Figure 7:**
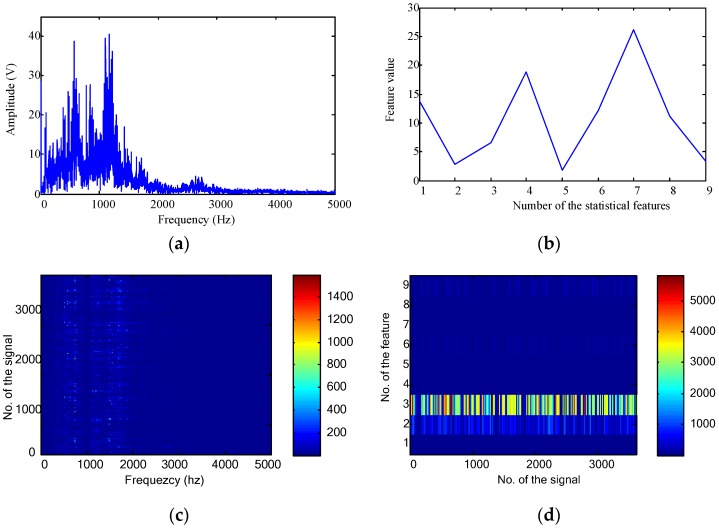
Frequency domain features for the gearbox fault diagnosis: (**a**) frequency domain representation of the first signal; (**b**) frequency domain statistical features of the first signal; (**c**) frequency domain representations of all the collected 3600 signals; and (**d**) frequency domain statistical features of all the collected 3600 signals.

Vibration signals were then transformed into the frequency domain. The frequency domain representation and statistical features for the first signal are shown in Figure 7a,b, respectively. As the sampling frequency was 10 kHz, the effective frequency band in Figure 7a is [0, 5000] Hz. However, there are only 4096 points for the temporal waveform. This means that there are only 2048 frequency points ranging between [0, 5000] Hz. For all the collected 3600 signals, their frequency domain representations and statistical features are shown in Figure 7c,d, respectively.

**Figure 8:**
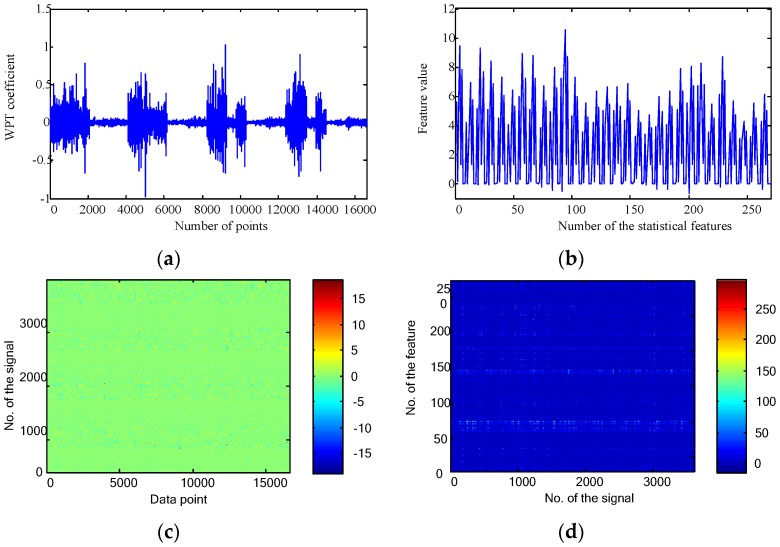
Time-frequency domain features for the gearbox fault diagnosis: (**a**) WPT representation of the first signal; (**b**) time-frequency domain statistical features of the first signal; (**c**) time-frequency domain representations of all the collected 3600 signals; and (**d**) time-frequency domain statistical features of all the collected 3600 signals.

For generating the time-frequency domain representations, the WPT was applied to decompose the raw data up to four levels. There are 2, 4, 8 and 16 nodes for each level. Put all the nodes together, the WPT presentation and statistical features are displayed in Figure 8a,b, respectively. As the length of the raw signal is 4096 points, numbers of data points for a node at the four levels are 2052, 1030, 519 and 264, respectively. In this way, the number of data points as shown in Figure 8a is 16,600. For the WPT, there are 30 nodes each of which has nine features. This generates 270 features for the first signal as shown in Figure 8b.

All the 3600 data have been disordered for the experiments. Among all the 3600 samples for each data, 2400 samples were random chosen as the training dataset *F*. To represent the statistical feature set *F*, we first applied the mon-layer GRBM with parameters as: number of the neurons in the hidden layer = 200, number of the learning epochs = 150, the initial learning rate = 0.001, its upper-bound = 0.001, and the weight decay = 0.005. As unsupervised learning of the GRBM does not have the classification function, a multi-class SVM classifier was applied to obtain the first fault diagnosis model (# 1 peer model). The reason for us to implement the model is to show the performance of the present deep learning. For # 1 peer model, the GRBM acts as the second feature representation tool (statistical features given by Equation (7) is the first one) for the vibration measurements. The outputs of the GRBM were fed into the SVM classifier. The supervised GDBM was subsequently applied for the same dataset *F* with parameters as: number of the neurons in the hidden layer 1 = 200, number of the neurons in the hidden layer 2 = 200, number of the pretraining epochs (for each constituent and the model) = 150, number of the fine-tuning epochs = 150, the initial learning rate = 0.001, its upper-bound = 0.001, and the weight decay = 0.005. In this way, we obtained the second fault diagnosis model (the proposed GDBM model). For comparison, the SVM classifiers for the original statistical features *M*(*p*,*q*), and the combination of *M*(*p*,*q*) and the GRBM representation were respectively developed as the third fault diagnosis model (#2 peer model) and the fourth one (#3 peer model). All the algorithms were realized using Matlab^®^. One may note that in this work we have not employed more “shallow” learning models such as the decision tree, the random forest, and the neural network. The reason is that the SVM has been proven the prominent representative which outperforms most of the “shallow” learning members.

With the trained models, the remaining 1200 samples (in the time, frequency, and time-frequency domains, respectively) were used to test the classification performances, which are displayed in Table 2.

From the diagnosis results shown in Table 2, it is clear that the classification rates for the time-frequency domain statistical features are higher (72.09% on average) than those for the time and frequency domains. This is due to the joint time and frequency representation of the WPT. When comparing the statistical features of the time and frequency domains, the time domain features are always the worst. Among all the models, deep statistical feature learning via the GDBM exhibits the best classification rate for the same data (62.58%, 91.75%, 95.17%, and 83.17% for the time, frequency, time-frequency domain statistical features, and the average, respectively). The best classification rate of 95.17% is seen with the GDBM model and time-frequency statistical features. Compared to supervised learning methods (e.g., the GDBM), the unsupervised GRBM displays the lowest classification rates (26.67%, 52.67%, 42.25%, and 41.33% for the time, frequency, time-frequency domain statistical features, and the average, respectively). Nevertheless, it should be noted that the GRBM used in this paper is an unsupervised algorithm, which shows that there is still some potential for fault diagnosis, if a fine-tuning procedure can be introduced for its learning process. As one of the most important “shallow” learning approaches, the SVM exhibited good classification results for the gearbox system. This result is similar to that of existing studies (e.g., [46]). When the GRBM representations are combined with the original statistical features *M*(*p*,*q*), a small increase in the classification rates can be seen (from 52.83% to 79.42% for the frequency domain, 69.50% to 78.42% for the time-frequency domain statistical features, and 61.05% to 64.56% on average). However, due to the “shallow” learning limit, it is very difficult to further improve the classification rate for the SVM. Our results indicate that deep statistical feature learning has the best performance for gearbox fault diagnosis. It should be noted that deep learning is much more time-consuming than classical learning methods.

### 4.2. Bearing Fault Diagnosis Results

**Figure 9:**
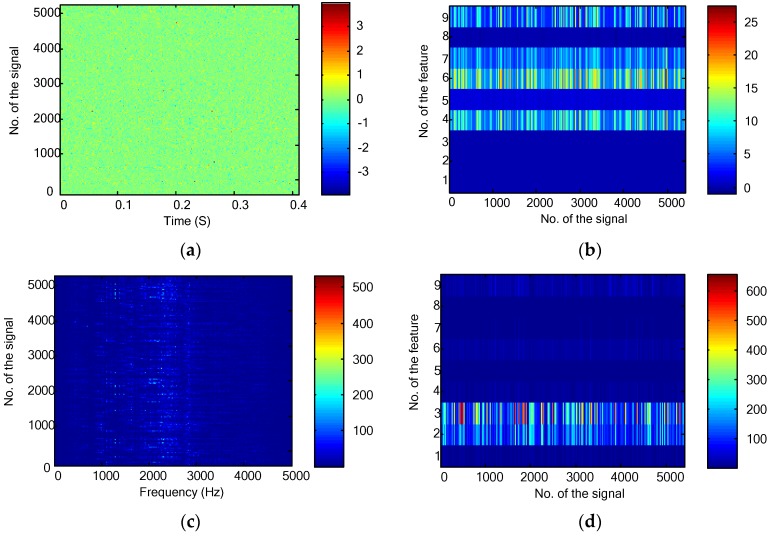

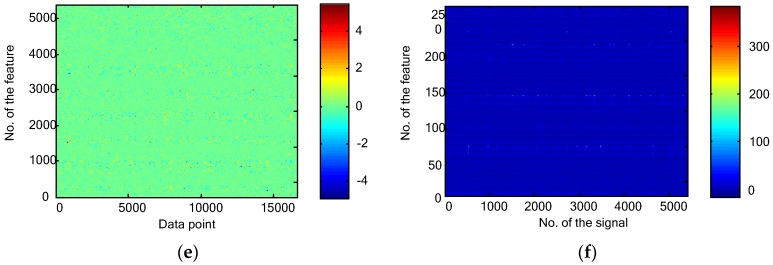
Bearing fault diagnosis experiments: (**a**) the time domain signals; (**b**) the time domain statistical features; (**c**) the frequency domain representations; (**d**) the frequency domain statistical features; (**e**) the WPT results; and (**f**) the time-frequency domain statistical features.

For the bearing fault diagnosis experiments, 5040 vibration signals and their statistical features in the time domain are plotted in Figure 9a,b. The Fourier transform were then used to generate the frequency data and their statistical features as shown in Figure 9c,d, respectively. The time-frequency representation produced by the 4-level WPT and their statistical features are shown in Figure 9e,f, respectively.

Of the 5040 samples, 3150 of the bearing system vibration signals were randomly chosen as the training dataset *F*. Similar modeling procedures to the gearbox fault diagnosis were repeated to develop the bearing fault diagnosis models. For comparisons, the same parameters are used in this subsection for the four models (*i.e*., No. 1: GRBM, No. 2: GDBM, No. 3: SVM, and No. 4: GRBM-SVM). After obtaining the trained models, the remaining 1890 samples (in the time, frequency, and time-frequency domains) were applied to test the classification performance for the bearing fault diagnosis. The results are displayed in Table 2.

A comparison of the feature performances in the different domains in Table 2 suggests that the time–frequency domain features exhibit the best performances (58.84%, 91.75%, 81.53% and 82.70% with the GRBM, GDBM, SVM and GRBM-SVM models, respectively), and the time domain features have the lowest classification rate (45.17% on average for all models). Compared with the gearbox fault diagnosis, the fault features for the rolling element bearings are more evident in the frequency domain, especially in the high frequency resonance band [47]. However, the model comparison results for the bearing fault diagnosis are almost same as those for the gearbox fault diagnosis. Among all the peer models, the deep statistical feature learning model (the GDBM) has the best classification rate (60.63% for the time domain, 87.57% for the frequency domain, 91.75% for the time-frequency domain, and 79.98% on average). This again validates the effectiveness of deep statistical feature learning for fault diagnosis in rotating machinery. Nevertheless, the improvement in fault diagnosis performance with deep learning is at the cost of complexity. The present GDBM is the most complex model with the largest number of parameters that must be estimated from the sample. It is an intrinsic drawback that “deeper” learning requires much more time than “shallow” learning does. As pointed out by LeCun *et al.* [48], the advent of fast graphics processing units (GPUs), which are convenient to program, allowed researchers to train deep networks 10 or 20 times faster. This indicates that parallel computation is helpful for reducing computation time. However, parallel computation is beyond the scope of this study. All the programs in this work were executed on a laptop. This resulted in much more computation time (hour-level) for the presented GDBM than its “shallow” counterparts (usually second- or minute- level on a laptop).

### 4.3. Remarks

Based on the fault diagnosis results as shown in the previous two subsections, one can see that the deep statistical feature learning holds the best classification performance comparing to the peer models. During the experiments, there were some very aberrant values (outliers) collected from the experimental setups, because the outliers are always unavoidable for real applications. It is obvious that the outliers may lead to deterioration of the fault diagnosis. However, we did not remove those outliers from the dataset, even if the removal of the outliers may increase the classification rates.

**Figure 10:**
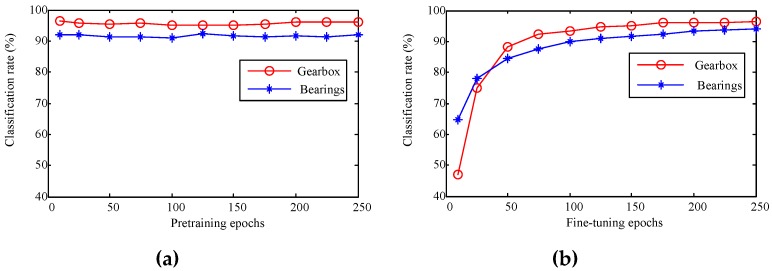
Relationship between the classification rate and the number of the modeling epochs: (**a**) classification rates v.s. pretraining epochs; and (**b**) classification rates *vs.* fine-tuning epochs of the time-frequency domain GDBM models.

It should be noted that the given parameters also play important roles to the GDBM model. As indicated by Cho *et al.* [35], the training procedure of the GDBM can easily run into problems without careful selection of the learning parameters. Upon determining the network structure for different layers, therefore, the learning epochs for the pretraining and the fine-tuning will be directly related to the classification performance. In this subsection we will discuss the influence of the epochs for the pretraining and the fine-tuning procedure. We first adjusted the number of pretraining epochs (for the GRBMs and the presented model) with all the other parameters fixed. Figure 10a plots the change of the fault classification rates in response to the increase of the pretraining epochs for the WPT features. For the fault diagnosis of both the gearbox and the bearing systems, the number of pretraining epochs does not influence the classification very much. This means that even for a small number (e.g., 10) of the epochs, the pretraining can achieve good effect.

The pretraining epochs were subsequently set at 150 to adjust the number of the fine-tuning epochs between 10 and 250. The fault classification rates for the two mechanical systems were displayed in Figure 10b. With the increase of the fine-tuning epochs, the classification rates for both experiments improve accordingly. For the gearbox diagnosis experiments, the improvement goes slowly after 125 epochs. As for the bearing systems, the classification rate increases still evidently before 200 epochs. Figure 10a,b prove that the deep statistical feature learning using the GDBM is not very sensitive to the learning parameters. Even though, a careful selection of the model parameters will be helpful in improving the fault pattern classification for the rotating machinery. This means that the proposed method has an essential improvement potential for the fault diagnosis of the rotating machinery.

**Figure 11:**
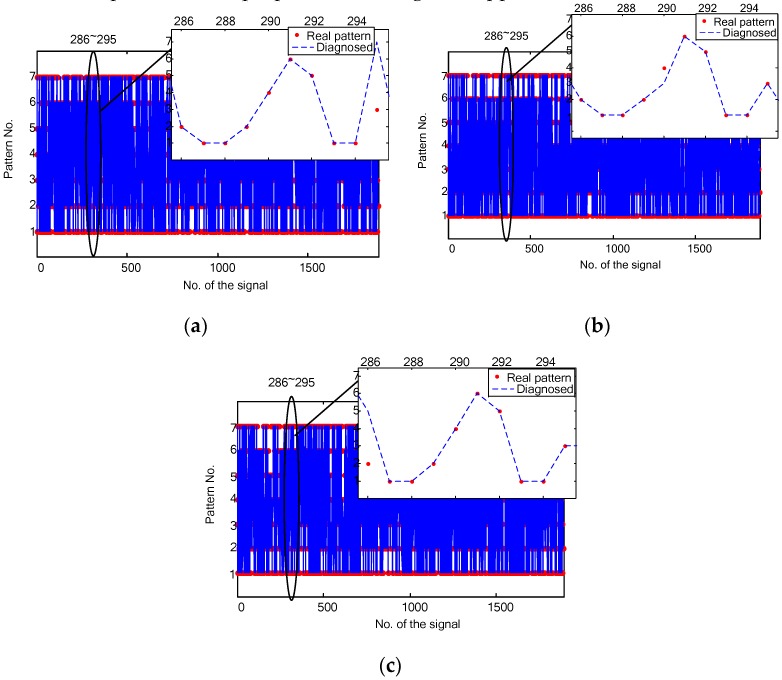
Bearing fault diagnosis results in different domains: (**a**) the time domain; (**b**) the frequency domain; and (**c**) the time-frequency domain.

Figure 11a–c plots the comparisons between the real fault patterns and the classified patterns for the bearing fault diagnosis in the time, the frequency and the time-frequency domains, respectively. It is shown that some signals are correctly classified by the proposed method in some domains, but are misclassified in other domains. Let’s take 10 vibration signals (#286~#295) of the bearing fault diagnosis as an example. The time domain diagnosis misclassified #295 signal, while both the frequency domain and the time-frequency domain obtained right classification. The frequency domain diagnosis misclassified #290 signal, but all the other two domains are correct. The time-frequency domain diagnosis misclassified #286 signal, while all the rest domains are right. If one considers the three domains simultaneously, therefore, all the 10 vibration signals (#286~#295) can be right diagnosed. This shows that the combination of the diagnosis results may contribute better classification rates. Though out of our scope in this paper, this discussion encourages us that further potentials can be explored for the proposed fault diagnosis approach.

## 5. Conclusions

In this paper, a deep statistical feature learning for vibration measurement has been proposed to diagnose fault patterns in rotating machinery. The statistical feature set was first extracted from the time, frequency, and time-frequency domains of the vibration signals. The real-valued RBMs were then stacked to develop a GDBM to accommodate statistical feature learning. Two typical rotating machinery systems (a gearbox and bearing test rigs), were constructed to validate the proposed approach, which was used for fault classification in the three-domain feature sets. The results show that deep statistical feature learning is capable of classifying fault patterns at higher rates than other models. Compared with the unsupervised GRBM, the SVM and the combined SVM and GRBM models, the deep statistical feature learning by the GDBM consistently had clearly better performances. This means that deep learning with statistical feature representation is a feasible update of conventional methods. The results also reveal that the statistical features in the time, frequency and time–frequency domains have different representation capabilities for fault patterns. Our further work will focus on optimizing the statistical features in different domains for different diagnostic applications.

## Figures and Tables

**Table 1:** Experimental configurations of different condition patterns.

Experimental Setup	Pattern Label	Component 1	Component 2	Load
Gearbox (component 1-pinion; component 2-gear)	A	Normal	Normal	zero, small, great
	B	Chaffing tooth	Normal	zero, small, great
	C	Worn tooth	Normal	zero, small, great
	D	Chipped tooth 25%	Normal	zero, small, great
	E	Chipped tooth 50%	Normal	zero, small, great
	F	Missing tooth	Normal	zero, small, great
	G	Normal	Chipped tooth 25%	zero, small, great
	H	Normal	Chipped tooth 50%	zero, small, great
	I	Normal	Missing tooth	zero, small, great
	J	Chipped tooth 25%	Chipped tooth 25%	zero, small, great
Bearing (component 1-bearing 1; component 2-bearing 2)	1	Normal	Normal	Zero, 1, 2 flywheel(s)
	2	Normal	Inner race fault	Zero, 1, 2 flywheel(s)
	3	Normal	Outer race fault	Zero, 1, 2 flywheel(s)
	4	Normal	Ball fault	Zero, 1, 2 flywheel(s)
	5	Outer race fault	Inner race fault	Zero, 1, 2 flywheel(s)
	6	Ball fault	Inner race fault	Zero, 1, 2 flywheel(s)
	7	Ball fault	Outer race fault	Zero, 1, 2 flywheel(s)

**Table 2:** Fault classification rates for the testing dataset (%), where N represents the device, d denotes the domain of the feature.

Device (*N*)	Domain (*d*)	Fault Diagnosis Model				
		#1 Peer	GDBM	#2 Peer	#3 Peer	Average ^a^
Gearbox	Time domain	26.08	62.58	60.83	35.83	46.33
	Frequency domain	52.67	91.75	52.83	79.42	69.17
	Time-frequency domain	45.25	95.17	69.50	78.42	72.09
	Average ^b^	41.33	83.17	61.05	64.56	62.53
Bearing	Time domain	18.52	60.63	59.58	41.96	45.17
	Frequency domain	39.95	87.57	80.74	82.91	72.79
	Time-frequency domain	58.84	91.75	81.53	82.70	78.71
	Average ^b^	39.10	79.98	73.95	69.19	65.508

^a^ the average value of the left four models; ^b^ the average value of the above three domains.
